# Challenges and Adaptations in COVID-19 Seroprevalence Surveys: Insights from Kinshasa, the Democratic Republic of the Congo

**DOI:** 10.3390/microorganisms14092056

**Published:** 2026-09-15

**Authors:** Benoit Mputu-Ngoyi, Angele Dilu-Keti, Paul Tshiminyi-Munkamba, Marc K. Yambayamba, Yannick Munyeku-Bazitama, Sheila Makiala-Mandanda, Antoine Nkuba-Ndaye, Steve Ahuka-Mundeke

**Affiliations:** 1 Département de Biologie Médicale, Cliniques Universitaires de Kinshasa, Kinshasa P.O. Box 127, Democratic Republic of the Congo; bmputungoyi2@gmail.com (B.M.-N.); shemakiala@yahoo.fr (S.M.-M.); amstev04@yahoo.fr (S.A.-M.); 2 TransVIHMI, Université de Montpellier, Institut de Recherche pour le Développement (IRD), Institut National de Santé et de Recherche Médicale (INSERM), 911 Avenue Agropolis, BP 64501, 34394 Montpellier CEDEX 5, France; angeledilu@gmail.com; 3 Département de Virologie, Institut National de Recherche Biomédicale, Kinshasa 01204, Democratic Republic of the Congo; doctapaul2010@gmail.com (P.T.-M.); ymunyeku@gmail.com (Y.M.-B.); 4 Département d’Epidémiologie et Biostatistiques, Ecole de Santé Publique, Université de Kinshasa, Kinshasa P.O. Box 127, Democratic Republic of the Congo; marc.yamba@unikin.ac.cd

**Keywords:** COVID-19, seroprevalence, household surveys, Democratic Republic of Congo, infodemic

## Abstract

Seroprevalence surveys constitute a critical tool for estimating population exposure to infectious agents, particularly during public health emergencies. In the context of the coronavirus disease 2019 (COVID-19) pandemic, such surveys revealed that the circulation of Severe Acute Respiratory Syndrome Coronavirus 2 in Africa had been largely underestimated. The validity of these surveys, however, depends not only on the reliability of diagnostic tests but also on operational conditions, which are frequently challenged by fear, misinformation, and logistical constraints. In the Democratic Republic of the Congo, the National Institute of Biomedical Research conducted two seroprevalence surveys in Kinshasa in 2020 on behalf the project “Appui à la Riposte Africaine à l’Epidémie de COVID-19” (ARIACOV). This study described the challenges encountered and the adaptive strategies employed during these surveys, in order to improve future investigations. The study population comprised 41 eligible field workers who participated in the 2020 ARIACOV seroprevalence surveys in Kinshasa, including 28 interviewers and 13 specimen collectors. Of these, 24 individuals (16 interviewers and 8 specimen collectors) initially confirmed their participation. Ultimately, 15 participants took part in the focus group discussions, comprising 11 interviewers (39.3% of the 28 eligible interviewers) and 4 specimen collectors (30.8% of the 13 eligible specimen collectors). We collected data through two focus groups held in a single session, using a pre-tested semi-structured guide. Discussions were audio-recorded and complemented by observational notes until empirical saturation was achieved. We applied deductive thematic analysis, following Braun and Clarke’s framework, to three predefined domains: transportation, survey procedures, and sample management. The application of systematic manual coding, combined with researcher triangulation, reinforced the rigor and enhanced the credibility of the findings. The findings highlighted community mistrust, driven by rumors, apprehension regarding blood collection, financial suspicions, and inadequate communication. In addition, logistical and transportation challenges significantly constrained survey feasibility. Nonetheless, a range of adaptive strategies were implemented, enabling the successful conduct of the surveys despite the pandemic context and limited resources. The participants’ narratives suggest that strengthening field workers’ training, improving transparency, and working alongside community health workers could contribute to enhance the effectiveness of serological surveys during health crises in resource-constrained environments.

## 1. Introduction

Seroprevalence surveys are designed to estimate the proportion of a population previously exposed to an infectious agent through the detection of specific antibodies, particularly in the context of outbreaks [1,2,3]. These surveys have become a critical public health tool for detecting silent transmission, identifying vulnerable subpopulations, and informing targeted prevention strategies [4,5,6].

During the Coronavirus Disease 2019 (COVID-19) pandemic, the importance of serosurveys has been highlighted in African settings, where virologic surveillance systems relying on molecular diagnostics, particularly Polymerase Chain Reaction (PCR), have demonstrated significant limitations [7,8,9,10,11]. Seroprevalence studies have revealed an underestimated circulation of Severe Acute Respiratory Syndrome Coronavirus 2 (SARS-CoV-2) in African settings [12,13,14,15,16,17,18]. However, the reliability of serological data depends not only on the analytical performance of the assays used, but also on the operational conditions [19,20].

The COVID-19 pandemic has further complicated sero-prevalence studies, particularly in contexts characterized by fear, misinformation, community mistrust toward health professionals and public authorities, and logistical constraints affecting field operations, all of which have required adaptation of data collection protocols to local realities [21,22,23,24]. These conditions, associated with social and economic impacts of epidemics, and existing vulnerabilities, can undercut both the feasibility and validity of serological investigations [25,26,27]. In this context, operational feedback from field workers (all trained personnel involved in community-based survey activities, including participant recruitment, informed consent, interviews, and specimen collection) represents a valuable source of insight for identifying limitations and opportunities to improve serological survey implementation in emergency settings [21,24]. Although rarely documented, such knowledge is essential to the success of these investigations [19].

In the Democratic Republic of the Congo (DRC), the first confirmed COVID-19 case was reported on 10 March 2020 in Kinshasa, the capital city [28]. The Congolese government promptly declared a state of emergency and established a national multisectoral committee to coordinate the pandemic response [29]. The implementation of public health measures, including social distancing, hygiene protocols, and country lockdown, had significant economic impacts [30,31,32], contributing to population mistrust regarding the existence and severity of the disease [24].

These measures also led to behavioral shifts, such as reduced social interactions and decreased reliance on public transportation [27]. As of 19 October 2020, following the first epidemic wave (March–July), the DRC officially recorded 11,078 confirmed cases and 303 deaths, with Kinshasa alone accounting for 74% of reported cases (8290 cases) [33].

These reports likely underestimated the true extent of viral transmission, given the limited access to testing and the high prevalence of asymptomatic and mildly symptomatic infections [34]. To address these gaps, the National Institute of Biomedical Research (INRB) conducted two household-based seroprevalence surveys between October and December 2020 (at the end of the first wave and during the second wave) as part of the ARIACOV study (Appui à la Riposte Africaine à l’Épidémie de COVID-19), among the general population of Kinshasa [35]. Both cross-sectional surveys were independently conducted by the same team of trained field data collectors.

Building on previously published serological surveys [17], this study documents the operational challenges faced during the two ARIACOV surveys and the strategies employed to address them, with the aim of deriving lessons to guide the design and optimization of future surveys in resource-limited, pandemic settings.

## 2. Methods

### 2.1. Study Design

We conducted a qualitative study to explore field workers’ experience of implementing the COVID-19 household seroprevalence surveys conducted in Kinshasa, the capital city of the DRC. We collected data through focus group discussions (FGDs) and semi-structured interviews conducted in April 2025 at INRB in Kinshasa. The study explored participants’ experiences of field implementation, operational challenges, community engagement, and lessons learned from conducting household seroprevalence surveys during the COVID-19 pandemic.

### 2.2. Data Collection Timeframe and Validity Issues in Qualitative Research

The timing of data collection represents a central methodological consideration in qualitative research. The 4.5-year interval between the ARIACOV surveys and the present study may raise concerns regarding recall bias. However, this time lag also provided an opportunity for more nuanced and contextualized reflections, enabling participants to articulate deeper insights into their experiences. Moreover, the use of a semi-structured discussion guide and the systematic pursuit of data saturation helped mitigate memory bias and reinforced the validity and robustness of the findings [36,37,38].

### 2.3. Selection and Recruitment of Study Participants

The study population comprised 41 field workers, including 28 interviewers (trained survey staff who administered standardized questionnaires and recorded participants’ responses), and 13 specimen collectors (trained healthcare personnel responsible for collecting, labeling, handling, and transporting biological specimens according to the study protocol and biosafety procedures), all aged 18 years or older, residing in Kinshasa, and having participated in the ARIACOV seroprevalence surveys conducted in 2020. Both male and female field workers were eligible for inclusion. The complete list of field workers who participated in the ARIACOV surveys served as the sampling frame. Potential participants were contacted by telephone to assess their availability and willingness to participate in the study. To ensure confidentiality and protect participants’ privacy, all personally identifiable and contact information was removed from the Supplementary Materials in accordance with data protection and participant anonymity principles (Table S1).

Of the 41 eligible field workers, 24 agreed to participate in the focus group discussions (FGDs), whereas 17 reported that they were unavailable (Table S1). This sample size was considered adequate to generate rich qualitative data while maintaining manageable group sizes that promoted active participation and dynamic interaction [38,39,40]. The recruitment strategy enabled the formation of FGDs that reflected the actual distribution of field roles during the ARIACOV surveys while ensuring the collection of diverse, context-specific perspectives, which were essential for understanding the complex dynamics of fieldwork conducted during the COVID-19 pandemic.

### 2.4. Organization and Composition of the Focus Group Discussions

Of the above-mentioned 24 individuals (16 interviewers and 8 specimen collectors) who initially confirmed their participation (Table S2), ultimately, 15 participants took part in the focus group discussions. Participants were assigned to the groups based on their role in the ARIACOV surveys, as well as their professional experience, age, sex, occupation, and survey area (urban or rural). To preserve the original fieldwork context, the composition of the FGDs intentionally maintained the interviewer-to-specimen collector ratio (approximately 2:1) observed during the ARIACOV surveys. Reproducing the original field team structure fostered authentic interactions and enabled participants to collectively reflect on their field experiences, operational challenges, and lessons learned during the seroprevalence surveys. This approach is consistent with methodological recommendations for focus group design, which emphasize the importance of clearly defined participant selection criteria and transparent justification of group composition to enhance the credibility and trustworthiness of qualitative findings [38,41,42].

### 2.5. Data Collection and Management Procedure

We used a semi-structured discussion guide, including open-ended questions and additional prompts to be used where necessary (Section S3). The guide was pre-tested to ensure clarity, relevance, and contextual appropriateness of the questions. Following the pretest, adjustments were made to simplify vocabulary, refine question order, and integrate prompts, thereby ensuring validity. These focus discussions were led by trained interviewers from our research team in French. Data collection proceeded iteratively until empirical saturation was reached, allowing the organization of a single session per group [43]. These two focus discussions were conducted the same day (Section S4), and they lasted approximately 110 min. Discussions were also recorded using a Sony dictaphone ICD-PX240, a digital recorder (Sony Corporation, Tokyo, Japan) (Section S4), with the permission of participants. A research assistant took detailed notes while capturing both the participants’ remarks and important non-verbal expressions.

### 2.6. Thematic Data Analysis Approach

We performed a deductive thematic analysis. The analysis used three predefined themes according to the field data collection process. These included: transportation of fieldworkers, the survey’s processes, and sampling and blood sample management. These themes were selected for their relevance in examining structural constraints, team dynamics, and technical procedures in the context of a public health emergency. They were embedded within an explicit conceptual framework informed by operational research and health systems performance models in crisis settings [44,45,46].

The analysis followed the six-phase methodological approach: 1. Familiarization with the data (repeated listening of audio recordings and in-depth reading of observational notes); 2. Initial coding (manual identification of meaning units related to the three predefined themes); 3. Sorting and grouping of codes (organization of excerpts into coherent subthemes); 4. Review of themes (validation of internal consistency and empirical saturation); 5. Definition and final naming of themes (synthesis and formulation of thematic findings); and 6. Report production (analytical writing of the findings, selection of illustrative excerpts, and alignment with the conceptual framework). In the first step, the researchers (BMN and PTM) manually and fully transcribed verbatim the recorded discussions independently to familiarize themselves with data, followed by the review of the third researcher (ADK). After that, a reference codebook for the set of two focus groups was created (Table S3). Transcripts were read several times in order to get a full understanding of them. These transcripts were then supplemented with notes taken by the research assistant (PTM) to produce a final document. The second step involved manual and directed coding and predefined themes, included the following steps: 1. Segmentation of the text into meaningful units relevant to the predefined themes; 2. Extraction and coding of these meaningful units; 3. Revision and classification of the initial codes into subcategories based on their similarities and differences; 4. Organization and aggregation of these subcategories into a broader categories; and 5. Development of topics that linked these categories to capture the underlying meanings. Throughout this process, codes were systematically compared across all data to ensure consistency and to identify patterns aligned themes.

### 2.7. Ethical Considerations

The study received approval from the Ethics Committee of the School of Public Health, University of Kinshasa (Approval Reference: ESP/CE/230/2024). To preserve confidentiality, participants were identified using neutral codes (P1, P2, etc.), and any information that could allow indirect identification was removed, in accordance with methodological recommendations [47,48]. Written informed consent was obtained from all participants. Prior to the commencement of the study, participants were informed of the objectives, the voluntary nature of their participation, the planned procedures, and their right to withdraw at any time without consequence.

## 3. Results

### 3.1. Number and Characteristics of Focus Group Participants

Of the 24 participants who had initially confirmed their participation, nine were ultimately unable to attend the focus group discussions because they were unavailable on the scheduled discussion dates. Consequently, 15 participants took part in the FGDs: nine in Group A and six in Group B. These included 11 interviewers (39.3% of the 28 eligible interviewers) and 4 specimen collectors (30.8% of the 13 eligible specimen collectors). Group A comprised seven interviewers and two specimen collectors, whereas Group B included four interviewers and two specimen collectors. The sociodemographic characteristics of all eligible participants, including both those who attended and those who did not attend the FGDs, are presented in Table S1. Among the nine non-attendees, five were interviewers and four were specimen collectors. The median age of participants who attended the FGDs was 36 years, compared with 32 years among those who agreed, but did not participate (Table S4).

### 3.2. Convergence Between Group A and B

Participants in both FGDs described similar challenges across sociocultural, logistical, and organizational domains, along with comparable adaptive strategies used to address these challenges.

#### 3.2.1. Sociocultural Barriers to Survey Participation

All fifteen FGD participants underscored major sociocultural barriers, including community mistrust, stigmatization, financial suspicions, field workers’ uncertainty, and fears related to blood collection.

##### Community Distrust

Twelve of the fifteen participants identified community mistrust as a dominant cross-cutting theme, expressed through several interconnected mechanisms.

First, all twelve participants described an infodemic—defined by the World Health Organization (WHO) as “too much information including false or misleading information in digital and physical environments during a disease outbreak’’—characterized by the circulation of contradictory rumors and conspiracy theories. According to participants, this information overload contributed to fear, mistrust, and rejection of the surveys. One participant illustrated this phenomenon as follows:

“There were too many rumors among the population during the COVID-19 pandemic that field workers wanted to buy corpses and inflate the number of cases to justify the government’s position and obtain money from the WHO.”

(P5, Group A)

Nine participants noted that these rumors gradually evolved into conspiratorial interpretations of the investigations, suggesting intentional harm directed against African populations:

“White people want to kill Africans like they did with AIDS to reduce their numbers and then steal our wealth. That’s why they manufactured COVID-19 in the lab to exterminate us.”

(P19, Group A)

One participant adopted a more nuanced position, attributing manipulation to government agendas rather than biomedical actors:

“You are just puppets of the government, which has hidden agendas, and it sends you to us to justify the use of the money that white people gave it.”

(P26, Group B)

Secondly, affiliation with the INRB was consistently identified as a trigger for immediate refusal within households, reflecting institutional rejection. Nine participants reported that simply mentioning the institution led to abrupt expulsion from households:

“Saying INRB was synonymous with refusal, because for the population, the INRB works with the Congolese government and the WHO, which is run by white people, to spread COVID-19 in Kinshasa so that everyone managing COVID-19 gets rich…”

(P24, Group B)

“As soon as we said we were from the INRB, they chased us away, saying we were there to give them COVID-19 and count them among the cases to make money, and that they wouldn’t get anything. Get out, we don’t want to be involved in your fake stories about COVID-19.”

(P7, Group A)

Ten participants emphasized that the population did not believe in the existence or severity of COVID-19, dismissing it as a mild and familiar illness akin to a cold or flu:

“People told us to stop talking about COVID-19, which doesn’t exist. There’s nothing like it in Africa. Look at how we live; if your COVID-19 really existed, we would all be dead like we see white people dying on France 24 in Italy, the USA, and other European countries.”

(P11, Group A)

“In the DRC, COVID doesn’t exist; it’s just our usual flu. We treat it with our medicinal plants, and it works. Since this dis-ease is killing white people in their own countries, they’re now trying to come and decimate Africans in collaboration with our easily corruptible leaders.”

(P8, Group B)

Finally, eight participants reported being explicitly discouraged in certain neighborhoods from mentioning or discussing COVID-19:

“People didn’t want us to talk about COVID. They said it was the white people who wanted to bother us with these stories because they’re dying in droves. We don’t have that problem.”

(P40, Group A)

##### Stigma and Social Isolation

Nine of the fifteen participants expressed participant’s fears of testing positive for COVID-19 and subsequently being subjected to isolation or stigmatizing funeral practices in light of rumors circulating during the previous Ebola outbreaks. This perception constituted a major barrier to participation in the surveys, as illustrated by one participant:

“If we test positive for COVID, we will be isolated, and if we die, we will be buried in a tarpaulin like the Ebola victims. The family won’t even be able to see the body to mourn.”

(P18, Group A)

##### Financial Suspicions

Ten of the twelve participants indicated that households perceived the surveys as a source of personal financial gain for field workers, thereby exacerbating existing socio-economic tensions. This perception was captured in the following statement:

“While we are stuck at home because of your lockdown measures, you continue to work, you will be paid, you will earn money; while we will earn nothing and continue to suffer.”

(P5, Group A)

##### Surveyor Uncertainty and Response Deficit

Seven of the twelve participants reported limited ability to respond to questions posed by households, particularly those concerning the transmission and prevention of COVID-19. This knowledge gap, reflecting the evolving nature of the pandemic, contributed to further erosion of community trust.

“In some households, we were asked, ‘If I am breastfeeding and I have COVID, can the baby catch it?’ As interviewers, we didn’t always have the answer. COVID was a new disease that took people by surprise and caused widespread panic. We were learning at the same time as everyone else. We were unable to provide reassuring answers to the public. We were in a difficult position.”

(P19, Group A)

##### Fears Related to Blood Collection

Ten of the twelve participants emphasized that concerns surrounding blood donation represented a major obstacle to survey participation. These concerns were structured around three interconnected dimensions: social and cultural perceptions of blood, questions regarding its use, and suspicions of manipulation or contamination.

- Cultural perceptions of blood as a vital and sacred substance

All ten participants indicated that, within local perceptions, blood was regarded as a precious substance intimately linked to life. This view rendered donation particularly sensitive, and often unacceptable, in the absence of a previously established relationship of trust. The following quotes illustrate this reluctance:

“For the public, blood is precious; you can’t just give it away.”

(P39, Group B)

“People were saying that blood is life, you don’t give it to strangers. We don’t know what they’ll do with it.”

(P24, Group B)

- Concerns regarding the purpose and volume of blood collected

Four of the ten participants noted that, beyond symbolic considerations, concrete questions arose concerning the actual use of blood, compounded by perceptions that the volume collected was excessive:

“When you take my blood, what are you going to do with it? Why are you taking so much blood like that? You’re suspicious with your COVID.”

(P8, Group B)

- Suspicion of deliberate transmission of COVID-19, forced vaccination, or mysterious manipulation of blood

Six of the ten participants reported that some households associated blood collection with attempts to inject COVID-19, clandestine vaccination, or occult practices:

“These people thought we were coming to inject them with the disease or the vaccine during the blood draw.”

(P9, Group B)

“Some said we were going to sell their blood for mystical purposes. Our religious beliefs don’t allow us to give our blood to dishonest people like you, who are spreading COVID in Kinshasa.”

(P8, Group B)

“The population was afraid, so afraid, uuuh, they said we were bringing COVID with us during the blood draw to make them sick.”

(P5, Group A)

#### 3.2.2. Logistical and Organizational Constraints

Eleven of the fifteen participants reported major logistical and organizational challenges that hindered the effective conduct of the surveys. These constraints directly affected team mobility, sample integrity, and individual safety, particularly in underserved or high-risk areas.

##### Challenges in Transporting and Delivering Field Materials

Eight of the eleven participants described significant physical obstacles in transporting inputs and accessing households, especially in mountainous regions and areas lacking road infrastructure. They emphasized that the burden of carrying coolers, sampling kits, and protective equipment over long distances caused considerable physical fatigue, thereby reducing operational efficiency:

“We had to climb a long mountain carrying our equipment and inputs by hand and in our backpacks. It was my first time climbing such a mountain. I was really tired and completely exhausted.”

(P8, Group B)

“In hard-to-reach areas, we carried coolers and bags filled with disinfectants, masks, and all the necessary equipment for sample collection, and then we had to walk long distances to reach households. It was difficult for us to conduct the surveys properly.”

(P7, Group A)

“We had to cross the river in dugout canoes or speedboats to get there. Sometimes, we took motorcycles, paying for our own transportation, to access the survey sites or to drop off samples at the INRB.”

(P18/P25, Group A; P24, Group B)

##### Late Returns and Curfew-Related Limitations

Six of the eleven participants reported that end-of-day logistics posed major challenges, particularly in ensuring the timely delivery of blood samples to the INRB. They explained that drop-offs frequently occurred late at night, during curfew hours, without transportation arrangements to guarantee field workers’ safe return:

“We returned to the INRB around 10:00 or 11:00 PM to drop off the samples, after spending a lot of time at various police stations during curfew. The vehicle remained at the INRB. Imagine the ordeal we endured on the way home under the same curfew and without transportation. It was difficult…”

(P7, Group A)

“The biggest challenge was how the person responsible for bringing the samples to the INRB late at night was going to get home during curfew. No vehicle was provided to transport the field workers. We had to find our own way home, often very late at night. And if it was a Saturday, we had to be at the INRB at 6:00 AM on Sunday to return to the field. It wasn’t easy… with the fatigue.”

(P9, Group B)

##### Interactions with Law Enforcement

Five of the eleven participants reported being required to present mission orders and, in some cases, to show blood samples at police checkpoints to justify their movements. These interactions caused significant delays and heightened psychological stress among field workers:

“We had to spend a lot of time at police checkpoints, talking with the police officers. We showed them our mission orders, but they insisted we show the samples before letting us through. After lengthy discussions, we showed them the samples…”

(P39, Group B)

##### Urban Insecurity

Seven of the eleven participants indicated that, in certain areas, the presence of gangs (Kuluna) created a climate of insecurity, limiting field workers’ access to households:

“The gangs in these neighborhoods thought we had a lot of money, especially when they saw us arrive in jeeps; the way they looked at us was frightening…”

(P24, Group B)

#### 3.2.3. Field Adaptation Strategies

In response to the sociocultural and logistical challenges encountered, all fifteen participants reported implementing a series of adaptation strategies aimed at strengthening community engagement, ensuring safety, and optimizing the conduct of surveys. Nine categories of adaptation strategies deployed in the field by the surveyors were identified.

##### Discourse Adjustment and Linguistic Reframing

To mitigate resistance associated with the use of the term COVID, thirteen of the fifteen participants reported reformulating their message using neutral language, such as “general health checkups” or “antibody studies.” They explained that this linguistic adjustment helped dissociate the surveys from anxieties related to diagnosis and fears of isolation:

“We told the population that the Ministry of Health sent us to conduct general checkups. You yourselves know that we live in a country where epidemics like cholera, typhoid fever, and malaria are frequent. We need to collect samples in order to conduct tests. We deliberately avoided mentioning COVID-19 for fear of being chased away.”

(P18, Group A)

“We had to clear up any ambiguity by explaining to people who wanted to know more about COVID-19 that this was not a screening test intended to quarantine people, but rather a study to identify the number of people in the population who had developed antibodies against COVID-19. These antibodies are very beneficial in protecting the population.”

(P26, Group B)

##### Engagement of Community Health Worker (CHW)

Thirteen of the fifteen participants emphasized that CHWs played a crucial role in legitimizing the surveys by facilitating access to households. They noted that the local credibility of the CHWs, as well as their strategic orientations, were essential levers for ensuring survey success:

“The CHW had anticipated our arrival in certain neighborhoods, and the households still gave us time to explain what the surveys entailed. After these explanations, some agreed to participate in the surveys, while others refused.”

(P39, Group B)

“The CHWs were key players; they negotiated with households on our behalf. Their presence helped to ease tensions within the population, especially when we were working with a well-known CHW in the neighborhood. We were heavily dependent on them, because without their involvement, I don’t know if we would have been able to recruit people. The population perceived us as coming to transmit COVID and inflate the number of cases so that our country would be classified among those with the highest number of COVID cases and obtain the funds promised by the World Bank, the IMF, and the WHO to countries affected by the pandemic.”

(P24, Group B)

“We worked with the CHW, who was very helpful in convincing the community. He was well-known in the neighborhood, and he guided us; we simply followed his instructions to maximize the chances of enrolling people in the surveys.”

(P25/P40, Group A)

However, two of the thirteen participants indicated that a CHW had refused to accompany them on the surveys, citing fear of community reprisals:

“The CHW was adamant, telling us he would not participate in the COVID-related surveys because he risked being killed by the residents of the neighborhood. He explained that when he had previously tried to talk to them about COVID, his house had been destroyed. I have conducted many surveys, but this was the first time a CHW refused to cooperate. That day, we only managed to enroll one household, and that was because a member of my team knew the family personally.”

(P8/P24, Group B)

##### Social Valorization and Scientific Framing

Nine of the fifteen participants reported presenting respondents as contributors to science and “heroes” of herd immunity, thereby valorizing their social utility and encouraging participation:

“We told them that COVID had wreaked havoc elsewhere, and that the WHO had predicted many deaths in Africa. But you are much stronger than COVID. You resisted it, and it didn’t kill you as feared. These words gave them a lot of courage, and they finally agreed to donate blood. We presented them as heroes, invincible against COVID, and it worked.”

(P5, Group A)

“We told the population that given how you live together, you had probably been in contact with COVID and you didn’t get sick or die. You probably developed antibodies collectively, what medicine calls herd immunity, a form of protection. We want to study this, and perhaps one day, thanks to your antibodies or your blood, we will be able to develop a vaccine against COVID. Hearing this, the population was pleased, thinking that a vaccine could be made from their blood and used to treat white people.”

(P11, Group A)

“We explained to the population the advantage of knowing their antibody status, which neutralizes the COVID virus, because it is possible to develop antibodies without getting sick. After hearing this type of explanation, some individuals were determined to participate in the studies in order to know their antibody status.”

(P24, Group B)

##### Strategic Targeting of Influential Household Members

Eleven of the fifteen participants indicated that they prioritized engaging household heads or influential figures to facilitate collective consent:

“When we entered a household, we identified the head of household, their representative, or a courageous member. We would engage in conversation with him, explaining the purpose of the surveys and describing the process, from signing the informed consent form to collecting samples. If the targeted individual agreed, they would then explain to the children and other house-hold members the importance of knowing their antibody status. In most households, the other members agreed to participate in the studies.”

(P39, Group B)

Furthermore, three of the fifteen participants reported using gender matching to overcome resistance expressed by the population:

“In my group, we implemented a strategy that involved, for example, allowing a female interviewer to speak with a hesitant or reluctant respondent when he had initially been approached by a male interviewer. This tactic helped us enroll individuals who were initially very reluctant.”

(P19, Group A)

##### Reassurance Through Physical Demonstration

To reduce apprehensions related to blood donation, four of the fifteen participants reported presenting sterile and unused equipment to reassure respondents regarding safety and hygiene conditions:

“We showed the population the empty, sterile syringe, still in its packaging and unused. Some individuals, seeing the quality of the collection equipment we had, agreed to participate in the surveys and consented to donate blood.”

(P8/P39, Group B)

“We showed the population clean, empty collection equipment to reassure them that we were not there to transmit COVID-19 to them, nor to administer the COVID-19 vaccine. This reassured the population and helped to dispel suspicions of contamination, secret vaccine injections, or even mysterious manipulation of their blood.”

(P5/P40, Group A)

##### Operational Flexibility and Instructional Adaptation

Fourteen of the fifteen participants indicated that they adjusted the instructions received during training to adapt them to local realities, prioritizing safety and feasibility:

“Upon our arrival in the field, and faced with the often-hostile attitude of the population towards us, we did not strictly follow all the instructions given during the training. If we had applied them rigorously, we risked not being able to conduct the surveys, as the population perceived us as too suspicious.”

(P19, Group A)

“During the training, we were told to follow the household selection procedure to comply with the sampling plan; however, given the realities encountered in the field, we asked the RECO (Regional Survey Coordinator) to guide us to households where they were known and where people were more likely to agree to participate in the surveys. In this case, even if we had calculated our sampling interval correctly, if the RECO told us not to enter because it was too dangerous, we disregarded the sampling interval.”

(P24, Group B)

##### Management of Barrier Measures and Behavioral Adjustment

Seven of the fifteen participants reported voluntarily refraining from wearing masks to avoid reinforcing community suspicions regarding COVID-19 transmission:

“We didn’t wear masks in the field so as not to frighten the population. Since they didn’t believe in the existence of COVID-19 and didn’t wear masks themselves, if we, perceived as those spreading COVID or administering the vaccine to kill people, wore masks, it would confirm their suspicion that we were there to administer the COVID vaccine…”

(P24, Group B)

##### Persuasion Techniques and Participatory Pedagogy

Ten of the fifteen participants emphasized the importance of patiently and repeatedly explaining the purpose of the surveys to foster trust and obtain informed consent:

“We were patient whenever a household gave us time to discuss the surveys. The team leader would begin by explaining; if the household remained unconvinced, another team member would rephrase the explanation in different terms. We remained patient and willing to address all their concerns. Thanks to these repeated clarifications, some households finally agreed to participate in the surveys.”

(P26, Group B)

“We really took the time to explain to households the benefits they could gain from these surveys. We answered their questions and did everything we could to establish trust first. Finally, some households agreed to participate in the surveys.”

(P39, Group B)

##### Material Incentives and Simulated Medical Consultations or Prescriptions

Fourteen of the fifteen participants reported distributing disinfectants and masks as part of their engagement strategy:

“We entered a compound with several households. In one household, the residents had refused to participate in the surveys. We went to the neighboring household, where the people listened to us and agreed to participate. We collected blood samples and then distributed disinfectants and masks. When the first household, which had initially refused, saw this, they called us back and asked to be enrolled as well…”

(P40, Group A)

Among them, four participants indicated that they had simulated medical consultations and/or prescriptions to strengthen credibility and encourage participation:

“In some households, when we introduced ourselves as health workers, the residents started describing their health concerns; for example, ‘I have gastritis,’ or ‘I have malaria.’ To build trust and encourage participation in surveys, we simulated a brief consultation or offered a prescription, such as an antacid or antimalarial drug. Following this interaction, households agreed to provide blood samples. Without such an approach, I’m not sure we would have been able to enroll them.”

(P11, Group A)

### 3.3. Points of Divergence Between FGDs A and B

While both focus groups highlighted several converging challenges and strategies, notable divergences also emerged, as summarized in Table 1.

## 4. Discussion

### 4.1. Community Distrust and Infodemic

According to the field workers, community distrust constituted a major obstacle to the implementation of serological surveys. Rumors and conspiracy theories fueled refusals to participate, and in some cases led to the expulsion of teams when their affiliation with the INRB was mentioned. Similar findings are reported by Doucet et al. (2022) [49], who demonstrated in Guinea that misinformation and the politicization of COVID-19 significantly undermined survey acceptability. Similarly, Raymond and Ward (2021) [50] emphasized that in low- and middle-income countries, the infodemic exacerbated distrust toward health programs. However, our data suggest a potential distinctive dimension by highlighting perceptions of institutional rejection linked to the INRB, a phenomenon that appears to be infrequently documented in the literature.

### 4.2. Education Level and Perception of the Disease

Field workers observed that the population often equated COVID-19 with a mild flu, reflecting limited access to information and inadequate health education. This observation aligns with UNICEF (2020) [51], which reported that the pandemic exacerbated educational inequalities in Africa and constrained the ability of communities to correctly interpret public health messages. Our findings build on this analysis by suggesting that limited understanding may have contributed to refusals to provide samples and misunderstandings of survey objectives, potentially limiting the reach and effectiveness of testing.

### 4.3. Logistical Constraints and Transportation

The field workers’ narratives revealed that traffic congestion, impassable roads, and reliance on alternative means of transport (e.g., motorcycles, canoes) slowed survey progress and compromised data quality. These observations corroborate the analyses of the High-Volume Transport Program (2021) [52], which documented how the pandemic disrupted mobility and logistics across Africa. Our findings suggest an additional operational consideration: field workers sometimes reported having to finance their own transportation, which may have increased their vulnerability and further constrained survey implementation.

### 4.4. Communication and Community Mediation

Field workers underscored the pivotal role of CHW in legitimizing the surveys and facilitating household access. This observation is consistent with Adebisi et al. (2021) [53], who highlight the importance of local communication and community engagement in building trust. Nevertheless, our findings suggest a point of divergence, with some community engagement actors reporting reluctance to cooperate because of fear of reprisals. This may indicate challenges affecting mediation mechanisms in crisis contexts and the effectiveness of community-based facilitation under conditions of heightened suspicion.

### 4.5. Financial Suspicion and Lack of Feedback

Field workers reported that households frequently perceived the surveys as a source of financial gain for the teams, thereby exacerbating existing socio-economic tensions. The absence of feedback on survey results further reinforced this distrust. These findings resonate with ethical debates on accountability and transparency in biomedical research in Africa, particularly the work of Mwaka et al. (2021) [54] and the H3Africa guidelines (2018) [55], which stress the importance of providing feedback to participants. Our findings suggest that the lack of feedback may have been associated with reduced participation in serological surveys, indicating that communication gaps could affect both trust and data collection.

### 4.6. Strengths of the Study

This study presents several notable strengths. It addresses a major public health issue in a pandemic context characterized by limited resources, while amplifying the voices of interviewers and specimen collectors whose experiences are rarely documented. The qualitative design, based on focus groups, a pre-tested semi-structured guide, systematic pursuit of saturation, and triangulation among researchers, provides methodological rigor. Furthermore, the deductive thematic analysis, structured around relevant operational themes, enables the identification of lessons directly applicable to the planning and optimization of future epidemiological surveys.

### 4.7. Limitations of the Study

Despite the richness of the data collected, this study presents several limitations that should be considered when interpreting the findings.

First, the relatively small sample size (*n* = 15) limits the transferability of the findings beyond the study population. Although 24 individuals met the inclusion criteria, only 15 participated in the study. The reason for non-participation among the remaining nine individuals was unavailability. It is therefore possible that those who participated differed systematically from those who did not, particularly in relation to the nature or intensity of their experiences. However, in such a small group, 15 participants can help achieve saturation.

Second, the study was conducted approximately five years after the household sero-surveillance surveys (October–December 2020), which may have introduced recall bias. This extended time lag may have affected participants’ ability to accurately recall events, and responses may be influenced by memory decay or subsequent public health experiences that occurred in the intervening period. While retrospective reflection may provide more considered narratives, the validity of such accounts should be interpreted with caution.

In addition, reliance on self-reported narratives introduces potential recall and social desirability biases. Participants may have underreported certain challenges or overemphasized the effectiveness of their coping strategies in describing their experiences.

Finally, the qualitative design of the study does not allow for any quantitative assessment of the magnitude of the obstacles encountered or the comparative efficacy of the strategies employed. The findings should therefore be interpreted as context-specific and exploratory rather than generalizable in statistical terms.

## 5. Conclusions

This study, based on firsthand accounts from ARIACOV interviewers and specimen collectors in Kinshasa, highlights three interdependent determinants of serological survey effectiveness in pandemic contexts: community perception, influenced by education levels and exposure to the infodemic; logistical constraints, which may affect the scope and quality of data collection; and communication and mediation mechanisms, whose fragility may undermine reliability in crisis settings.

Grounded in field experiences, these findings indicate that strengthening field workers’ training, enhancing community communication, and improving transparency of results are means to build trust and support the effectiveness of serological surveys during health emergencies.

## Figures and Tables

**Table 1:** Divergent findings between focus group discussions A and B.

Dimension	FGD A	FGD B
Security context	Limited exposure to violence; emphasis on discretion	Active presence of gangs (Kuluna); identification of red zones
Physical fatigue	Strong emphasis on physical exertion and energy depletion	Mentioned, but less central to participants’ narratives
Media influence	Impact of international media coverage on perceptions	Dominance of local rumors and social media narratives
Relational approach	Individualized and gender-sensitive engagement strategies	Predominantly collective and group-based interaction dynamics
Medical framing	Simulated medical consultations used in some households to build trust	No mention of medical framing or simulated consultations

## Data Availability

The raw data supporting the conclusions of this article will be made available by the authors on request.

## Supplementary Materials

The following supporting information can be downloaded at: https://www.mdpi.com/article/10.3390/microorganisms14092056/s1, Table S1. Characteristics of field workers invited to the study; Table S2. Distribution of twenty-four participants across FGDs A and B; Table S3. Codebook of field challenges and adaptive strategies during ARIACOV surveys (FGDs A and B); Table S4. General characteristics comparison between respondent and absent field workers; Figure S1. Documenting the conduct of FGD A; Figure S2. Documenting the conduct of FGD B; Figure S3. Dictaphone.

## Abbreviations

AIDS	Acquired Immune Deficiency Syndrome
ANRS	Agence Nationale de Recherche sur le SIDA
ARIACOV	Appui à la Riposte Africaine contre l’Epidémie de COVID-19
CEAs	Community Economic Advisors
CHW	Community Health Worker
COVID-19	Coronavirus Disease 2019
DRC	Democratic Republic of the Congo
ESP	Ecole de Santé Publique
FGD	Focus Group Discussion
IMF	International Monetary Fund
INRB	Institut National de Recherche Biomédicale
SARS-CoV-2	Severe Acute Respiratory Syndrome Coronavirus 2
PRISME	Plateforme de Recherche Internationale en Santé Mondiale en République Démocratique du Congo
WHO	World Health Organization
