# Supplementary material for: Challenges and Adaptations in COVID-19 Seroprevalence Surveys: Insights from Kinshasa, the Democratic Republic of the Congo
Source: Microorganisms. 2026 Sep 15;14(9):2056. doi: 10.3390/microorganisms14092056 (PMC13608894; doi:10.3390/microorganisms14092056)
Supplement: Supplementary file 1 [file microorganisms-14-02056-s001.zip › microorganisms-4343293-supplementary.pdf]

Challenges and adaptations in COVID-19 seroprevalence surveys: insights from Kinshasa, the Democratic Republic of the Congo

Supplementary Materials

Section S1: Table S1. Characteristics of field workers invited to the study

Order	Participant	Age	Sex	Occupation	Role during surveys	Participation in surveys prior to ARIACOV	Survey area	Participation status
1.	P12	27 Years	F	Psychologist	Interviewer	6	Urban	Agreed but did not participate in FGDs
2.	P15	35 Years	F	Nurse	Interviewer	3	Rural	Agreed but did not participate in FGDs
3.	P20	40 Years	F	Nurse	Interviewer	5	Urban	Agreed but did not participate in FGDs
4.	P21	40 Years	M	Physician	Interviewer	5	Urban	Agreed but did not participate in FGDs
5.	P27	23 Years	F	Computer scientist	Interviewer	4	Rural	Agreed but did not participate in FGDs
6.	P29	22 Years	M	Laboratory Technician	Specimen collector	0	Urban	Agreed but did not participate in FGDs
7.	P31	33 Years	M	Laboratory Technician	Specimen collector	2	Rural and Urban	Agreed but did not participate in FGDs
8.	P32	27 Years	M	Laboratory Technician	Specimen collector	3	Rural	Agreed but did not participate in FGDs
9.	P35	36 Years	F	Laboratory Technician	Specimen collector	8	Urban	Agreed but did not participate in FGDs
10.	P5	36 Years	M	Computer scientist	Interviewer	2	Rural	Participated in FGDs
11.	P6	36 Years	M	Physician	Interviewer	2	Rural	Participated in FGDs
12.	P7	29 Years	M	Physician	Interviewer	2	Rural and Urban	Participated in FGDs
13.	P8	36 Years	F	Physician	Interviewer	2	Urban	Participated in FGDs
14.	P9	36 Years	F	Physician	Interviewer	2	Rural and Urban	Participated in FGDs
15.	P11	42 Years	F	Physician	Interviewer	5	Rural	Participated in FGDs
16.	P18	44 Years	F	Physician	Interviewer	3	Rural and Urban	Participated in FGDs
17.	P19	41 Years	M	Physician	Interviewer	6	Urban	Participated in FGDs
18.	P24	37 Years	M	Physician	Interviewer	1	Rural and Urban	Participated in FGDs
19.	P25	36 Years	F	Marketing agent	Interviewer	3	Rural	Participated in FGDs
20.	P26	32 Years	M	Physician	Interviewer	0	Rural and Urban	Participated in FGDs
21.	P30	33 Years	F	Laboratory Technician	Specimen collector	0	Urban	Participated in FGDs
22.	P34	46 Years	F	Laboratory Technician	Specimen collector	7	Rural	Participated in FGDs
23.	P39	30 Years	M	Laboratory Technician	Specimen collector	2	Rural	Participated in FGDs
24.	P40	33 Years	M	Laboratory Technician	Specimen collector	2	Rural and Urban	Participated in FGDs
25.	P1	27 Years	F	Physician	Interviewer	2	Rural	Reported being unavailable for the FGDs
26.	P2	40 Years	M	Physician	Interviewer	1	Urban	Reported being unavailable for the FGDs

27.	P3	33 Years	M	Nurse	Interviewer	5	Urban	Reported being unavailable for the FGDs
28.	P4	24 Years	F	Nurse	Interviewer	0	Rural	Reported being unavailable for the FGDs
29.	P10	36 Years	F	Physician	Interviewer	4	Rural and Urban	Reported being unavailable for the FGDs
30.	P13	27 Years	M	Nurse	Interviewer	1	Rural	Reported being unavailable for the FGDs
31.	P14	39 Years	M	Nurse	Interviewer	7	Rural	Reported being unavailable for the FGDs
32.	P16	23 Years	F	Nurse	Interviewer	2	Urban	Reported being unavailable for the FGDs
33.	P17	30 Years	M	Nurse	Interviewer	2	Rural and Urban	Reported being unavailable for the FGDs
34.	P22	35 Years	M	Physician	Interviewer	5	Rural	Reported being unavailable for the FGDs
35.	P23	26 Years	F	Nurse	Interviewer	0	Rural	Reported being unavailable for the FGDs
36.	P28	31 Years	F	Physician	Interviewer	0	Urban	Reported being unavailable for the FGDs
37.	P33	32 Years	M	Laboratory Technician	Specimen collector	3	Rural	Reported being unavailable for the FGDs
38.	P36	45 Years	F	Laboratory Technician	Specimen collector	2	Rural and Urban	Reported being unavailable for the FGDs
39.	P37	31 Years	M	Laboratory Technician	Specimen collector	1	Urban	Reported being unavailable for the FGDs
40.	P38	29 Years	F	Laboratory Technician	Specimen collector	1	Rural and Urban	Reported being unavailable for the FGDs
41.	P41	24 Years	M	Laboratory Technician	Specimen collector	4	Rural and Urban	Reported being unavailable for the FGDs

Note: *Field workers were identified using neutral codes (P1, P2, etc.). Their names, telephone numbers, and any other information that could potentially allow their identification have been omitted from this table in order to preserve anonymity.*

FGDs: Focus Group Discussions; M: male; F: female; ARIACOV: Appui à la Riposte Africaine à l'Epidémie de Covid-19

.

Section S2: Table S2. Distribution of twenty-four participants across FGDs A and B

Focus group discussion	Participant	Age	Sex	Occupation	Role during surveys	Number of participations in surveys prior to ARIACOV	Survey area	Availability at the FGD session
**FGD A**	P5	36 Years	M	Computer scientist	Interviewer	2	Rural	Participated in FGDs
	P6	36 Years	M	Physician	Interviewer	2	Rural	Participated in FGDs
	P7	29 Years	M	Physician	Interviewer	2	Rural and Urban	Participated in FGDs
	P11	42 Years	F	Physician	Interviewer	5	Rural	Participated in FGDs
	P18	44 Years	F	Physician	Interviewer	3	Rural and Urban	Participated in FGDs
	P15	35 Years	F	Nurse	Interviewer	3	Rural	Agreed but did not participate in FGDs
	P19	41 Years	M	Physician	Interviewer	6	Urban	Participated in FGDs
	P25	36 Years	F	Marketing agent	Interviewer	3	Rural	Participated in FGDs
	P29	22 Years	M	Laboratory Technician	Specimen collector	0	Urban	Agreed but did not participate in FGDs
	P31	33 Years	M	Laboratory Technician	Specimen collector	2	Rural and Urban	Agreed but did not participate in FGDs
	P34	46 Years	F	Laboratory Technician	Specimen collector	7	Rural	Participated in FGDs
	P40	33 Years	M	Laboratory Technician	Specimen collector	2	Rural and Urban	Participated in FGDs
**FGD B**	P8	36 Years	F	Physician	Interviewer	2	Urban	Participated in FGDs
	P9	36 Years	F	Physician	Interviewer	2	Rural and Urban	Participated in FGDs
	P12	27 Years	F	Psychologist	Interviewer	6	Urban	Agreed but did not participate in FGDs
	P20	40 Years	F	Nurse	Interviewer	5	Urban	Agreed but did not participate in FGDs
	P21	40 Years	M	Physician	Interviewer	5	Urban	Agreed but did not participate in FGDs
	P24	37 Years	M	Physician	Interviewer	1	Rural and Urban	Participated in FGDs
	P26	32 Years	M	Physician	Interviewer	0	Rural and Urban	Participated in FGDs
	P27	23 Years	F	Computer scientist	Interviewer	4	Rural	Agreed but did not participate in FGDs
	P30	33 Years	F	Laboratory Technician	Specimen collector	0	Urban	Participated in FGDs
	P32	27 Years	M	Laboratory Technician	Specimen collector	3	Rural	Agreed but did not participate in FGDs
	P35	36 Years	F	Laboratory Technician	Specimen collector	8	Urban	Agreed but did not participate in FGDs
	P39	30 Years	M	Laboratory Technician	Specimen collector	2	Rural	Participated in FGDs

Note. *The participants in FGDs A and B retained their original codes.*
FGDs: Focus Group Discussions ; M: male; F: female; ARIACOV: Appui à la Riposte Africaine à l'Epidémie de Covid-19

Section S3: Discussion guide

Introduction and icebreaker

Welcome, everyone.

I am Dr. Benoit MPUTU, serving as the moderator for this session. Dr. Paul will act as the note-taker.

We sincerely thank you for participating in this study entitled "Challenges and adaptations in conducting seroprevalence surveys during COVID-19 pandemic: insights from Kinshasa, the Democratic Republic of the Congo." This study refers to the two ARIACOV surveys conducted between October and December 2020, in which you participated either as interviewers or sample collectors.

This focus group will last approximately 60 to 90 minutes and will be conducted in a single session. It will explore three thematic areas, each comprising several open-ended questions intended to stimulate discussion.

All contributions are valuable-there are no right or wrong answers.

Participants are encouraged to speak freely and respectfully.

The moderator will facilitate turn-taking.

The note-taker will document all verbal contributions using copying paper.

The session will be audio-recorded using a voice recorder (Dictaphone); participants are kindly requested to speak one at a time to ensure clarity.

To begin, kindly introduce yourself by stating your first or last name, your role during the survey, and a memorable or surprising experience related to your work during the pandemic.

Follow-up: How many years of experience do you have in conducting surveys of this nature?

Theme S1: Staff and equipment transportation

Q1. How was transportation organized between INRB and survey sites?

Q2. How was moving performed between health zones and health areas?

Q3. How was moving performed from a household to another?

Q4. What types of logistical challenges did you faced while moving?

Follow-up

- Were these related to vehicle availability, road conditions, interactions with other road users, or welcoming in specific areas?

Staff logistics

- How did field teams organize themselves during the survey?

Q5. Did you face any specific challenges related to the pandemic? Please describe.
Were their delays or absences due to transportation issues?

Material logistics

- How was transportation of supplies (e.g., equipment, sampling kits) managed?

Follow-ups

- Was this process affected by the health crisis?
- How did the situation influence transportation from storage facilities to field sites?
- Did you have to adapt procedures or improvise?

Theme S2: Field survey implementation

Q6. How did interactions with household respondents take place during the pandemic?

Q7. Did the pandemic influence household selection procedures within health areas? Were any criteria or protocols adapted?

Q8. How were households approached to participate in the study?

Follow-up

- Did social distancing measures alter your approach?
- What concerns did households express regarding interviewers during the pandemic?

Example follow-up

- Can you share specific situations or examples?

Comparison follow-up

- Did household reactions vary by profile, health area, or health zone?

Q9. What do you perceive as the main reasons for household refusal or reluctance to participate during the pandemic?

Q10. What strategies did you use to engage hesitant or resistant households? Were any of these strategies' pandemic-specific?

Q11. In your view, how well was the training adapted to the pandemic context?

Theme S3: Blood sample collection and field storage prior to INRB delivery

Q12. What challenges did you face during blood collection?

Q13. How did participants react to blood sampling during or after the pandemic? Can you provide concrete examples?

Q14. Were there refusals linked to fear or specific beliefs? What reasons, rumors, or misconceptions did you encounter?

Q15. How did you address these challenges? What strategies were implemented?

Q16. Were you adequately equipped to manage these challenges?

Q17. How was sample transport to the laboratory organized?

Q18. How did the pandemic affect this phase of your work? Were there delays, procedural deviations, or storage-related issues?

Conclusion

- Based on today's discussion, what do you consider the most significant impacts of the pandemic on survey implementation?

What key insights do you retain from your own experience and from what others have shared?

Section S4: Images documenting the conduct of FGDs A and B, and Dictaphone

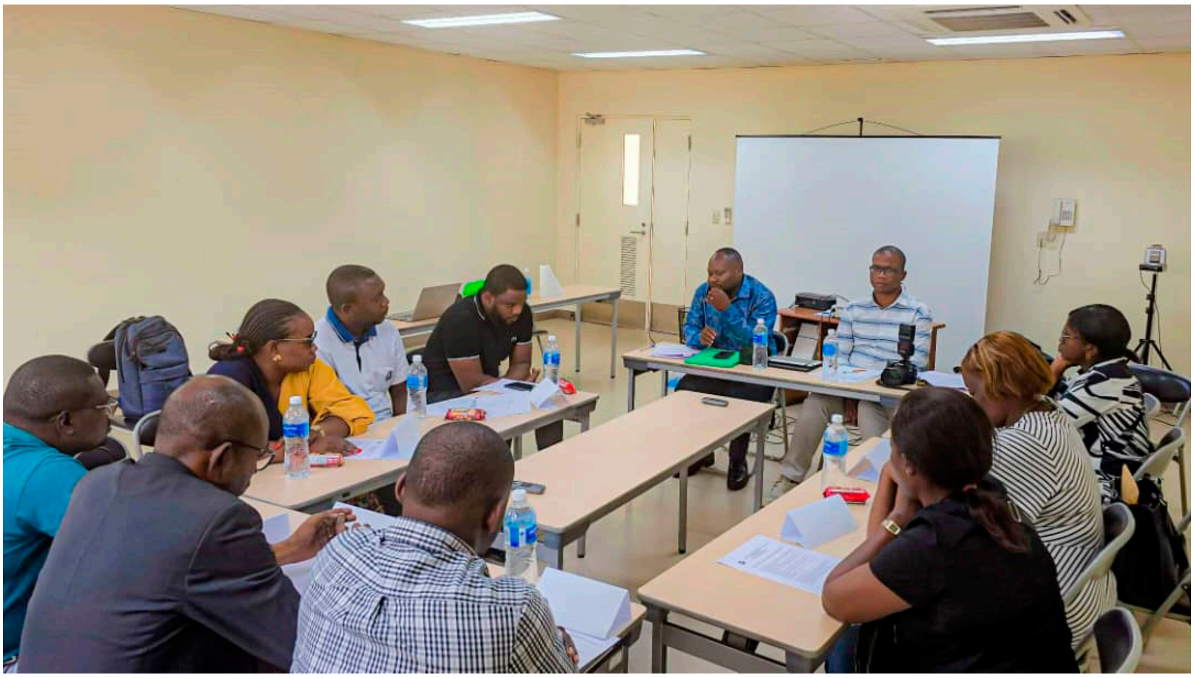

Figure S1. *Documenting the conduct of FGD A*

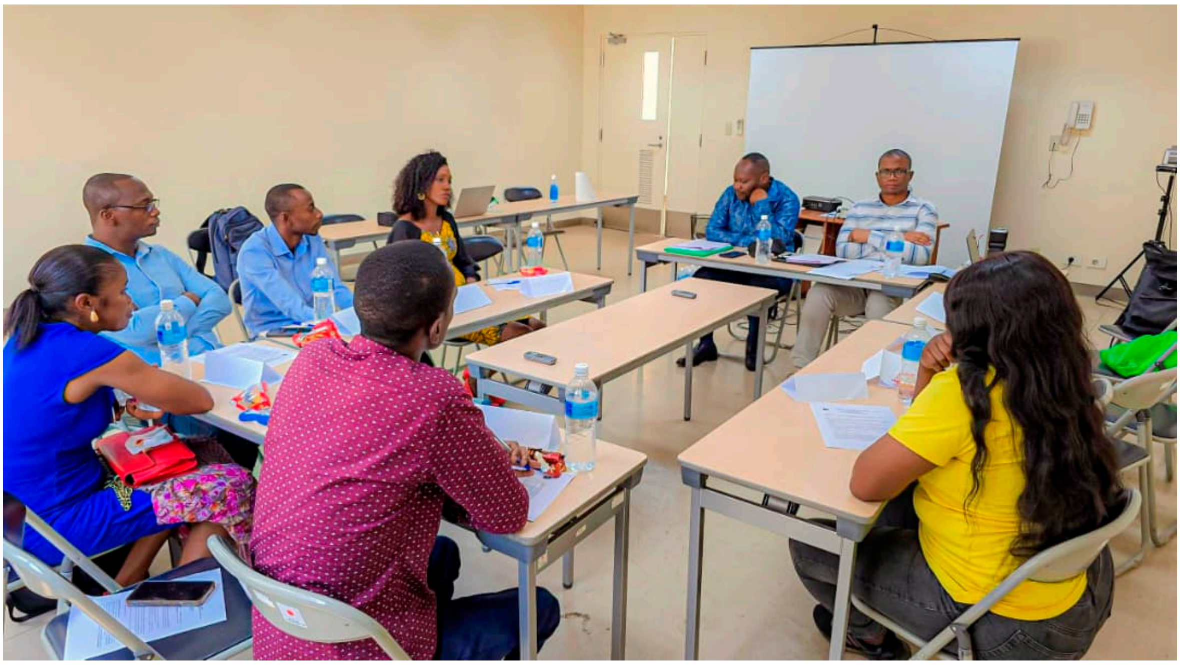

Figure S2. *Documenting the conduct of FGD B*

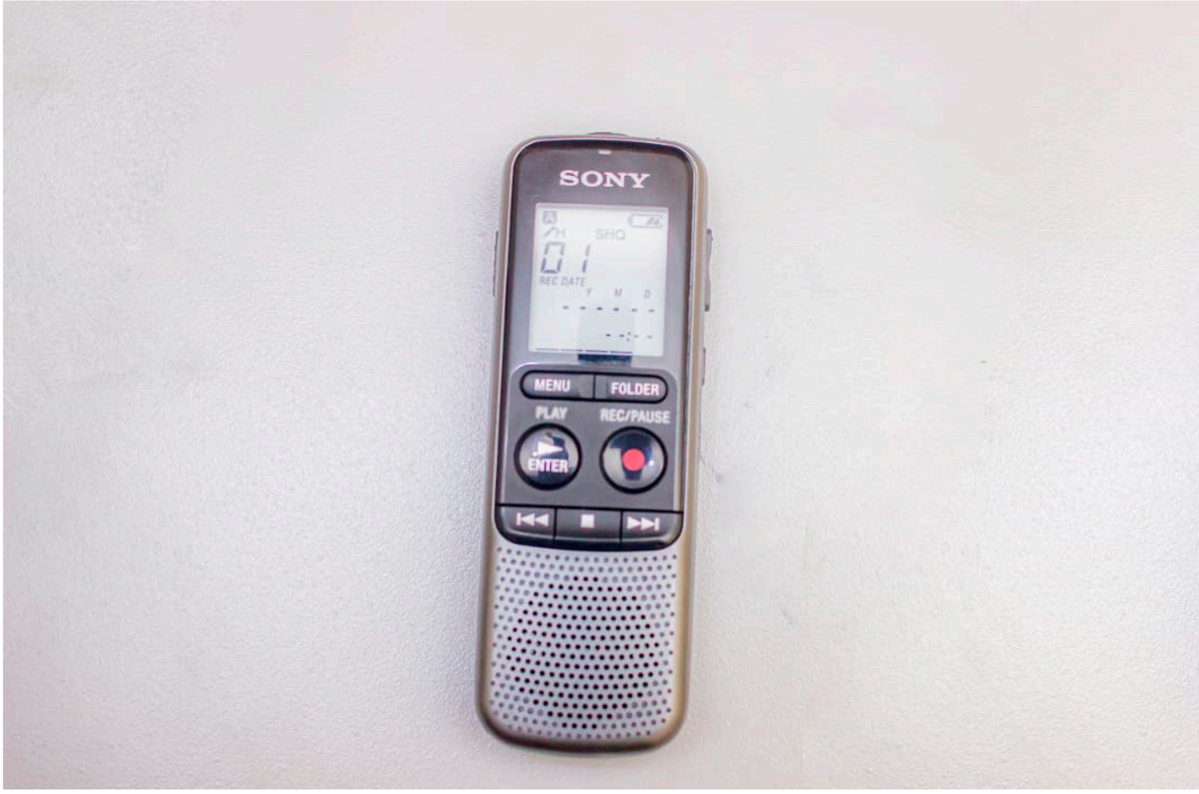

Figure S3. *Dictaphone*

Section S5: Table S3. Codebook of field challenges and adaptive strategies during ARIACOV surveys (FGDs A and B)

Code	Definition	Inclusion Criteria	Illustrative Quote	Sub-theme	Theme
COVID Denial	Refusal to believe in the existence or severity of COVID-19	Statements denying the reality or impact of the pandemic	*"COVID doesn't exist, there's nothing …"* (P11, Group A); *"It's just a flu …"* (P8, Group B)	Disease denial	Community Mistrust
COVID Rumors	Circulation of false information about COVID-19	References to media, social networks, animated or conspiratorial discourse	*"I heard rumors, white people made up their COVID stories to exterminate Black people and make money. They started telling people: you must get vaccinated or you'll die of COVID. Just like with AIDS."* (P19, Group A)	Rumors and infodemic	
Survey Refusal Profile	Households refusing to participate or to discuss the topic	Excerpts describing "refusal," hostility, or non-participation	*"They didn't want us to talk about COVID…"* (P18/P40, Group A and P4/P39, Group B)	Household profile-based refusal	
Survey difficulties	Difficulties encountered by Interviewers in obtaining responses	Mentions of interruptions, non-responses, or evasive answers from respondents or scientific Interviewers	*"We asked… if it was needed, I said: I don't know."; "They didn't answer us, they didn't give us the answers."* (P5/P11, Group A)	Interviewer uncertainties	
Generalized fear reaction	Any segment expressing generalized fear or apprehension equivalent to fear of vaccination	"They were afraid! They thought we were injecting something else, or feared being vaccinated against COVID without their consent." (P8, Group B)	*"They were afraid! They believed we had come to infect them with COVID or to vaccinate them against COVID without their consent."* (P5/P11, Group A)	Generalized fear	
Rumors distrust interaction	Suspicion fueled by COVID-19 rumors, perceived as justification for refusal to participate	Mentions of "rumors," "fear," "mistrust," or "suspicion" describing refusal	*"Those people thought we wanted to kill them by giving them the vaccine!"* (P3/P39, Group B)	Rumors and mistrust	
Blood concept	Mention of blood as sacred or mystical, making donation to a stranger impossible	Mentions of "blood is life," "you can't just give it away"	*"…blood is life, we don't give it."* (P24, Group B)	Blood conception	

Participant interest	Reflections on personal benefit (what's in it for us?) underlying participation	Excerpts on perceived benefit or lack of "accountability" in studies	*"In their view, people asked themselves: what do we gain?"; "When people saw the surveys being conducted, they disappeared; it made the survey more complicated."* (P26, Group B)	Participant interest-seeking	
Conspiracy perception	Ideas of conspiracy or hidden agenda fueling mistrust	Segments mentioning "puppets," "government's hidden agenda," or future use of samples	*"Puppets… if the government has hidden agendas, they'll use you…"* (P26, Group B)	Conspiracies and mistrust	
Blood concept blood	Blood as sacred or mystical, making donation to a stranger impossible	Mentions of "blood is life," "you can't just give it away"	*"…blood is life, we don't give it."* (P24, Group B)	Cultural conception of blood	
Conspiracy Reasoning	Beliefs that the survey conceals vaccination intentions (forced control, population reduction)	Excerpts on "hidden agenda," "pharmaceutical conspiracy," "lockdown to enrich a few"	*"If the government has hidden agendas… they'll use you…"* (P26, Group B)	Conspiracies and plot	
Religious refusal	Refusal motivated by religious beliefs	Explicit references to religious beliefs and prohibition of donation	*"Their religious beliefs didn't allow it."* (P8, Group B)	Refusal due to religious beliefs	
Question's reaction	Repeated questions about the survey's objectives	Excerpts where people ask questions like "why," "what," "how many ml," "what are the results for"	*"When you take my blood, what are you going to do with it?"; "Why so much blood?"* (P8, Group B)	Questioning and concerns	
INRB rejection	Rejection of institutions, especially the name INRB	Denial of access, avoidance of the word INRB	*"Saying INRB was synonymous with refusal…"* (P24, Group B) ; *"People reacted violently against INRB, we were chased away."* (P19, Group A)	Mistrust toward INRB	

Blood fear	Fear related to blood collection and its use	Refusal to give blood, fear of manipulation or sale	*"You want to sell our blood to white people to make money."* (P8, Group B) ; *"They thought we wanted to inject them with the disease."* (P9, FGD B)	Fear of blood collection	
COVID-19 stigmatization	Fear of being identified as COVID-positive and quarantined	Mentions of quarantine, confinement like Ebola	*"They'll bury you in a tarp like Ebola."* (P18, Group A)	Stigmatization and isolation	
Information level distrust	Household resistance linked to low understanding of COVID-19 and its assimilation to flu or exaggeration	Include statements denying COVID-19's existence, equating it to flu, or expressing exaggeration of the pandemic	*"People told us to stop talking about COVID-19, that there was no COVID here in Africa. Look how we live; if your COVID really existed, we'd all be dead."* ; *"They said COVID-19 is a made-up disease to scare us."* ; *"In DRC, COVID doesn't exist; white people lied to us."* ; *"They made it up to scare us."*; *"They made it up to kill us."* ; *"They made it up to scare us, to kill us, to exterminate us."*; *"They made it up to kill us, to exterminate our medicinal plants."* (P18, Group A and P5/P8/P24, Group B)	Health literacy (regional education level)	
CHW collaboration refusal	Situation where a community health worker refuses to participate or is perceived as participating under constraint due to fear of COVID-related stigma	Include any mention of a CHW refusing to participate or being forced to participate due to fear of COVID-related stigma	*"A community health worker (CHW) refused to work with us in the field, stating that his house had been burned down when he was raising awareness among the population about the reality of COVID-19. It was the first time I had seen a CHW refuse to participate in a survey."* (P8/P24, Group B)		
Supply transport constraints	Obstacles related to the movement of equipment and teams	Fatigue, breakdowns, difficult terrain, traffic jams	*"It was a mountain… we had to climb it carrying our supplies by hand."* (P8, Group B)	Transport difficulties	Logistical and Organizational Constraints

Checkpoint Roadblock Delays	Delays due to roadblocks and inspections	Checkpoints, mission orders, sample inspection	*"We had to show the mission order… otherwise they wouldn't let us pass. The police made us wait a long time before letting us through."* (P9, Group B)	Police controls	
Physical fatigue equipment handling	Physical strain from carrying equipment and samples	Mentions of fatigue, hardship, carrying coolers	*"Carrying the cooler and bags full of supplies on our backs… it was quite exhausting."* (P7, Group A)	Physical hardship	
Return logistics sample courier	Constraints linked to the transporter being left alone at INRB – no vehicle to return home after sample drop-off	Mentions of the "obligation for the escort" to return alone and late, without a vehicle	*"The person who brings the samples to INRB – how will he get home… with the curfew, no vehicle, that was the major difficulty."* (P9, Group B)	Escort's return	
Exposure urban insecurity	Presence of gangs or dangerous areas	References to gangs (Kuluna), red zones, fear	*"The gangs thought we had a lot of money, and the way they looked at us was a bit scary!"* (P24, Group B)	Urban insecurity	
Late return logistics sample collection	Late return to INRB after sample collection, causing fatigue and scheduling constraints	Explicit mentions of late return, hardship of bringing samples back late at night	*"Returning to INRB to drop off samples when we finished late… the vehicle stayed at INRB, with the curfew, getting home… it was exhausting."* (P7, Group A)	Late return logistics	
Police interactions Roadblocks	Interactions with law enforcement at checkpoints: identity checks, sample and mission order verification causing delays	Excerpts mentioning "checkpoints", "agents, police" requesting documentation and use of mission order to pass	*"At other checkpoints, the police forced us to show the samples… otherwise they wouldn't let us pass, even though we showed the mission order."* (P39, Group B)	Roadblocks and police checks	
Impassable roads river crossings	Difficulties in traveling between INRB, health zones, and households (traffic, fuel, canoes, motorcycles)	Include statements about terrain, self-funded costs, transport incidents	*"To get to the survey site, we had to cross water… take a motorcycle… canoes."* (P25, Group A) ; *"The vehicle… had no fuel. So we had to wait a long time before heading out. That day, we arrived very late."* (P7, Group A) ; *"We also took speedboats and canoes to cross the river."* (P18, Group A)	Site access	

Mobility risks field hazards	Exposure of teams to police checks, urban threats (Kuluna), or accidents	Include statements about arrests, threats, gang escorts, road incidents	*"We were arrested… even though we showed the mission order. We had to call the supervisor who resolved the situation by phone."* (P11, Group A) ; *"We worked in Masina and Kinsenso, we had to hide the car… the Kuluna chief escorted us."* (P5/P11, Group A and 26, Group B) ; *"Our driver hit another vehicle… we were detained until it was repaired."* (P7, Group A)	Safety and mobility	
Designation sample transport responsible	Designation of a person (team leader or Specimen collector) responsible for bringing all samples to INRB	Any passage mentioning that "a single person" is designated for transport	*"We assigned the team leader to bring the samples to INRB with the driver… But the vehicle had to stay at INRB… with the curfew, getting home was difficult."* (P9, Group B)	Designation of a single transporter	
Use neutral language Field communication	Substitution of the word "COVID" with neutral terms	Use of "general check-up," "malaria," etc.	*"We often said general check-up…"* (P18, Group A) ; *"We always said we came for an interview…"* (P5, Group A) ; *"We said… you have typhoid, you have malaria… to reassure the population…"* (P18, Group A and P24, Group B)	Discourse reformulation	
Community relay involvement	Involvement of CHWs to introduce and reassure	Mentions of CHWs as facilitators, guides, mediators	*"In certain neighborhoods, the CHW participated in our arrival and the population opened the door for us to conduct surveys…"* (P39, Group B)	Support from community relays	Field adaptation Strategies
Social salorization participants	Presenting households as heroes or contributors to science	References to immunity, exemplary role, contribution to vaccination	*"We had to portray the population as heroes who COVID did not reach or kill…"* (P5, Group A)	Participant valorization	
Material reassurance through demonstration	Presentation of empty syringe, cleaning, transparency	Presentation of empty syringe, cleaning, transparency	*"We showed them the syringe: there's nothing inside…"* (P8/P39, Group B and P5/40, Group A)	Material demonstratio n	

Targeting household decisionmakers	Targeting the household head to facilitate family adherence	References to "household head," "entity leader," ripple effect	*"If the household head accepts, he explains to the children, he agrees to participate in the surveys…"* (P39, Group B)	Targeting the household head or influential person	
Gender-Based interaction strategies	Use of gender complementarity, adaptation by sex	References to mixed teams, female teams	*"A very hesitant female team, when we send a female team, people accept…"* (P5, Group A)	Gender affiliation	
Immunity-based persuasive narratives	Argumentation around collective and individual immunity	Excerpts discussing collective immunity, individual protection, contribution to knowledge	*"We had to say you are immune…"* (P5, Group A) ; *"We said you are immune, you had COVID, you have antibodies, you are healthy, the survey is less likely."* (P11, Group A)	Collective immunity narrative	
Hostile interactions refusal entry	Aggressive attitudes or refusal of entry, sometimes advised by CHW for safety reasons	Segments where CHW forbids entry or expresses hostility ("do not enter," "it's very dangerous")	*"The CHW forbade us from entering, said it was very dangerous for us. We didn't go in. So in the field, we followed the CHW's instructions given during training."* (P39, Group B)	Hostility and refusal	
Sampling protocol adjustments safety	Need to follow and rely on CHW to avoid dangerous areas	References to "no sampling," orientation or prohibition of a household by CHW for safety reasons	*"We often didn't follow the sampling. We followed what the CHW told us to properly conduct the surveys."* (P39, Group B)	Respect for sampling and safety	
Clarification diagnostic research intent	Clarification that this is a detection study (not research or surveillance)	Excerpts mentioning "not screening," "study to see if you developed antibodies"	*"We had to clear up the misunderstanding in people's minds by saying we were not doing screening, but a study to find out who had antibodies."* (P26, Group B)	Distinction between diagnosis and research	

Trust building community relays	Trust established through community relay involvement	Segments presenting CHW's role as key ("relied heavily on them…")	*"CHWs were really key people… we relied heavily on them…"* (P24, Group B) ; *"Without the CHWs' presence, I don't know if we could have enrolled the population who were very afraid of COVID…"* (P19, Group B)	Trust climate via CHW	
Instructional flexibility sampling procedures	Adaptation of sampling instructions based on real situations	Mentions of "sampling" being set aside or modified per CHW instructions	*"Regarding sampling… even if the household is well calculated, in the field if the CHW says 'don't enter that household,' we didn't go in."* (P24, Group B)	Sampling flexibility	
Adaptation barrier measures local context	Adaptation of barrier measures to avoid frightening the population	Explicit mentions of modified instructions to avoid alarming the population	*"We didn't wear masks in the field to avoid scaring people: we approached those who came out of the house."* (P24, Group B)	Abandonment of barrier measures	
Demonstrative reassurance strategies	Familiarization with survey materials (masks, demonstrations) and cleaning to show safety	Any passage where "materials are shown," "unused items are cleaned," to reassure	*"We had to show clean and empty materials so the population would realize we weren't there to transmit COVID or vaccinate them against COVID…"* (P5, Group A)	Reassurance through demonstration	
Use material incentives encourage participation	Mentions of distributing disinfectants and masks as leverage	Mentions of distributing disinfectants and masks as leverage	*"When a household didn't want to participate in the survey and we gave disinfectants to the one who accepted, they called us: 'Hey, we accept now.'"* (P40, Group A)	Material incentives	
Pragmatic flexibility field protocols	Adaptation of instructions based on field realities	Adjustments, circumvention, pragmatic adaptation	*"In the field, we didn't apply the instructions to the letter…"* (P19, Group A) ; *"We didn't follow the sampling, we told the CHW, take us where people trust you so we can enroll them with the CHW…"* (P24, Group B)	Instruction flexibility	
Iterative explanation clarification	Re-explanation by several team members for households	exchanges, explanatory relay	*"One member explains, another follows up and agrees to answer their questions. We spent more time than expected…"* (P26/P39, Group B)	Explanatory repetition	

Respectful insistence patience	Respectful insistence until a response is obtained	Mentions of insistence, perseverance, patience	*"Through clarification and explanations, some households eventually opened the door and we enrolled them…"* (P26, Group B)	Patience and persistence	
Simulated medical consultation trust building	Prescription of medication to encourage participation	Prescription of medication to encourage participation	*"In some households, we did a small consultation or medical prescription to gain people's trust… no narachol, we enrolled them…"* (P11, Group A)	Simulated prescription	

Section S6: Table S4. General characteristics comparison between respondent and absent field workers

Characteristics	FGDs participants n=15	Agreed but did not participate in FGDs n=9	Unavailable for the FGDs n=17
Age, median (years)	36	33	31
Sex (M/F)	8/7	4/5	9/8
Ratio Interviewers/Specimen collectors	11/4	5/4	12/5
Number of participations in surveys (median)	2	4	2
Survey areas			
Rural	6	3	7
Urban	3	5	5
Rural and Urban	6	1	5

FGDs: Focus Group Discussions; M: male; F: female
